# Emerging Role of USP8, HMGA, and Non-Coding RNAs in Pituitary Tumorigenesis

**DOI:** 10.3390/cancers11091302

**Published:** 2019-09-04

**Authors:** Daniela D’Angelo, Marco De Martino, Claudio Arra, Alfredo Fusco

**Affiliations:** 1 Istituto di Endocrinologia ed Oncologia Sperimentale—Consiglio Nazionale delle Ricerche (CNR) c/o Dipartimento di Medicina Molecolare e Biotecnologie Mediche, Università degli Studi di Napoli “Federico II”, 80131 Naples, Italy; 2 Dipartimento di Psicologia, Università della Campania, 81100 Caserta, Italy; 3 Struttura Semplice Dipartimentale Sperimentazione Animale, Istituto Nazionale dei Tumori, Fondazione Pascale, 80131 Naples, Italy

**Keywords:** pituitary tumors, epigenetics, HMGA, USP8, non-coding RNAs

## Abstract

Two novel molecular mechanisms with a driver role in pituitary tumorigenesis have been recently identified. They are (a) mutations in the Ubiquitin-Specific Protease 8 (USP8) gene in corticotroph tumors and (b) overexpression of the *HMGA1* and *HMGA2* genes in most of the pituitary tumors. Moreover, deregulated expression of the non-coding RNAs has been very frequently observed in this neoplasia. The aim of this review is to better elucidate the role, the mechanisms, and the possible clinical impact of these novel alterations in the development of pituitary neoplasia.

## 1. Introduction

Tumors of pituitary gland (PT), found in 20% of the population, are recurrent brain tumors exhibiting different behaviors [1]. The great majority of PT are benign neoplasms, usually named pituitary adenomas (PA), that are composed of adenohypophyseal cells. However, some of them are invasive, displaying several recurrences and resistance to standard therapies. Hence, whereas PA classification based on the released hormones is widely accepted, the classification based on their invasiveness, recurrences, and resistance to treatment is still controversial [1,2] since the PA features are highly heterogeneous and complex. 

In the recent past, a classification of a subgroup of atypical adenoma based on the expression of the proliferation markers (i.e., Ki-67 or p53) was proposed, but it was unable to estimate the resistance to therapy or recurrence [3]. Another effort was to classify PAs in five grades, pointing out the tumors with high risk of recurrences, but, unfortunately, this proposal also failed [4]. 

Very recently, it has been proposed by the International Pituitary Pathology Group Club, supervised by Prof. J. Trouillas, that the tumors of adenohypophyseal cells may be named pituitary neuroendocrine tumors (PitNETs), using a nomenclature that has been widely accepted for other neuroendocrine tumors [5]. Consistently, several PitNETs, although not metastatic, similarly may induce a strong morbidity. In fact, some PitNETs, producing an excess of hormones, may lead to dramatic clinical syndromes, such as acromegaly or Cushing’s disease (CD), that are characterized by mood disorders, sexual dysfunction, infertility, obesity and disfigurement, visual disturbances, hypertension, diabetes mellitus, and accelerated heart disease [6]. Furthermore, many PitNETs increase in size quickly, causing symptoms specific of an intracranial mass that comprise headache, visual-field disturbances, and failed production of anterior pituitary hormones [6].

The molecular mechanisms that lead to the pathogenesis of these common tumors are still largely unexplored. In particular, the genetic alterations that have been extensively detected in other human tumors, such as mutations in Ras, B-RAF, and p53 genes, have been found just in rare human PitNETs. 

It is noteworthy that few cases of PitNETs arise due to familial or hereditary syndromes [7]. There are two groups of familial PitNETs: (i) An isolated group, with the presence of just tumors of pituitary gland and (ii) a syndromic group, characterized by several neoplasias. 

The most frequent genetic alterations causing the familial isolated pituitary adenoma (FIPA) are the aryl hydrocarbon receptor-interacting protein (AIP) gene mutations [8], and the duplication of *GPR101* gene that leads to X-linked acrogigantism (XLAG) [9]. AIP is a 330 amino acid co-chaperone protein acting as a tumor suppressor. AIP has several interactors and its tumorigenic role is linked to the deregulation of cAMP pathway [10], a key role pathway in somatotroph tumorigenesis. Interestingly, lower AIP expression correlates with invasiveness [11] and somatostatin responsiveness expression in somatotroph adenomas [12]. 

XLAG is a newly discovered cause of growth hormone (GH) overabundance. It is caused by a de novo microduplication of X chromosome (Xq26.3). Although the duplicated chromosomal area comprises four different genes, only the *GPR101* one has been found overexpressed in pituitary tissues. GPR101 was identified as an orphan Gs protein-coupled receptor whose endogenous ligand and function remain still unknown. However, its activation induces an increase of cAMP levels [13,14]. 

The majority of hereditary syndromes are linked to either MEN-1 (associated with genetic mutations of the tumor suppressor gene MEN-1, which codes for the transcriptional repressor menin that regulates several cell-cycle proteins such as p27^kip1^ and CDK4), Carney’s complex (CNC) (associated with genetic alterations of the tumor suppressor gene PRKAR1A, leading to excessive cAMP signaling), and McCune–Albright syndrome (where activating mutations have been found in the *gsp* oncogene sequence) [7]. Recently, a MEN-4 syndrome, caused by a loss-of-function of *CDKN1B* gene, coding for p27^kip1^, has been identified [15]. These patients are often hit by GH-secreting PitNETs together with other tumors such as parathyroid adenomas, renal angiomyolipomas, neuroendocrine cervical carcinomas, papillary thyroid carcinomas, and gastric carcinomas [15]. Moreover, germline mutations in *DICER1* gene, coding for the cytoplasmic enzyme that induces the maturation of hairpin precursor miRNAs into functional microRNAs (miRNAs) gives rise to the so-called DICER1 syndrome that is characterized by the development of several neoplasias, including pituitary blastoma [16].

Noteworthy, several sporadic PitNETs could be little masses with a long-lasting growth rate: These hormonally inactive PitNETs are generally found “incidentalomas” by radiographic analyses or at post-mortem examination [17]. Moreover, sporadic tumor growth is likely stimulated by hormones that regulate the physiological pituitary activity, and by growth factors that have been involved in standard fetal pituitary progression [18]. Interestingly, some murine models of pituitary tumors have underlined the differences between mice and humans [19]. Thus, the events that lead to human pituitary tumorigenesis represent an interesting paradigm to study the development of human tumors of endocrine system, likely shedding new light on non-endocrine tumors. 

## 2. Methods and Objectives

A systemic literature search was performed using the following keywords: “pituitary tumor” and “deubiquitinase”, “pituitary tumor” and “HMGA”, and “pituitary tumor” and “non-coding RNA”. Medline/PubMed was carried out for our primary search. In this review, we focus on the role of Ubiquitin-Specific Protease 8 (USP8) mutations, HMGA protein overexpression, and deregulation of non-coding RNA expression in pituitary tumorigenesis since these findings have been reported quite recently and their deep analysis may be, in our opinion, of particular interest for the researchers involved in this field.

## 3. Somatic Mutations in Ubiquitin-Specific Protease 8 (USP8) Gene in ACTH-Secreting PitNETs 

About 10% of PitNETs secrete ACTH and induce CD, a disease that is frequently linked to some injuries, such as diabetes, hypertension, secondary infections, osteoporosis, and an augmented risk of death for cardiovascular injuries [20].

The gravity of the clinical symptoms depends on the levels of hormonal hypersecretion, the sensitivity of glucorticoid receptors, and time of the exposure [20]. In some cases, larger ACTH-secreting PitNETs are related to augmented possibility of local injuries, such as visual impairment and hypopituitarism [20]. However, except for some evident radiological signs of invasiveness, there are no predictors of poor surgical outcomes, and then markers of tumor growth and invasiveness would be of great clinical relevance. 

The most important finding in pituitary tumorigenesis in the recent years is represented by the somatic mutations in the *USP8* gene, coding for a deubiquitinase that is a member of the ubiquitin-specific protease family. In fact, two distinct research groups have recently identified missense mutations located in the exon 14 of the *USP8* gene [21,22]. In one of these studies, *USP8* mutations were detected in 6 out of 17 ACTH-secreting PitNETs patients [21]. In another study, analogous mutations were found in 8 out of 12 patients [22]. Then, the same authors analyzed USP8 mutations in 108 ACTH-secreting PitNETs patients: 67 cases (62%) were found positive for USP8 mutations [22].

**Figure 1:**
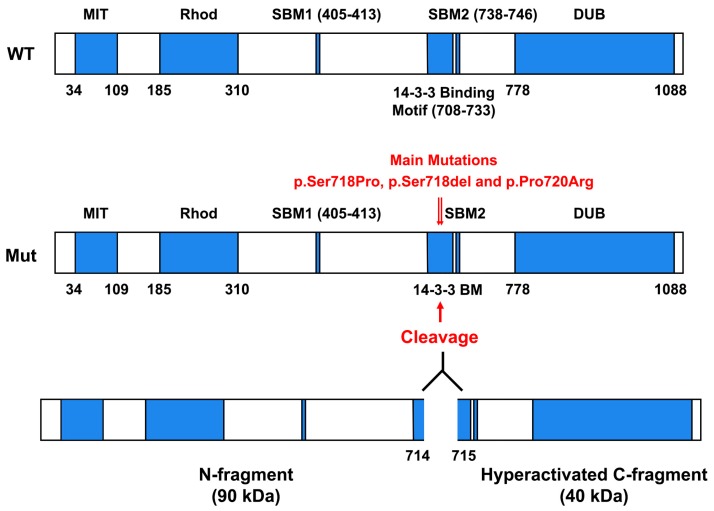
Ubiquitin-Specific Protease 8 (USP8) hotspot mutations in ACTH-secreting pituitary neuroendocrine tumors (PitNETs). Normally, 14-3-3 proteins bind to the 14-3-3 binding motif (BM) of USP8 Wild-Type (WT) protein when the residue Ser718 is phosphorylated, decreasing USP8 deubiquitinase activity. The most frequent mutations are p.Ser718Pro, p.Ser718del, and p.Pro720Arg, and these mutants completely lose the capability to bind to 14-3-3 proteins. Intriguingly, all these USP8 mutants, after a proteolytic cleavage, generate two fragments. The 40 kDa fragment, or C-fragment, contains the hyperactivated deubiquitinase catalytic domain.

As shown in the Figure 1, the USP8 protein is composed of five different domains:

(1) Microtubule-interacting and trafficking domain (MIT) (aa 34 to aa 109) that is required for efficient abscission at the end of cytokinesis, together with components of the ESCRT-III complex.
(2) Rhodanese-like domain (Rhod) (aa 185 to aa 310).
(3) SH3-binding motif (SBM) (aa. 405 to aa 413).
(4) 14-3-3-binding motif (14-3-3 BM) (aa 708 to aa 733). Vertebrate USP8 protein sequences have a well-conserved 14-3-3 BM, which consists in RSYSSP sequence. In particular, 14-3-3 proteins, a protein family composed of seven isoforms in humans, are important regulatory proteins that can bind to a consensus sequence, RSXpSXP (where X represents any amino acid and pS represents phosphorylated Serine), controlling the functions and the cellular compartmentalization of several 14-3-3 BM-carrying proteins [23]. Intriguingly, it has been found that the binding of 14-3-3 protein to 14-3-3 BM of murine USP8 strongly decreased its deubiquitinase activity on ubiquitinated Epidermal Growth Factor Receptor [24].
(5) Deubiquitinase catalytic domain (DUB) (aa 778 to aa 1088) that removes the conjugated ubiquitin molecules from the target proteins.

**Figure 2:**
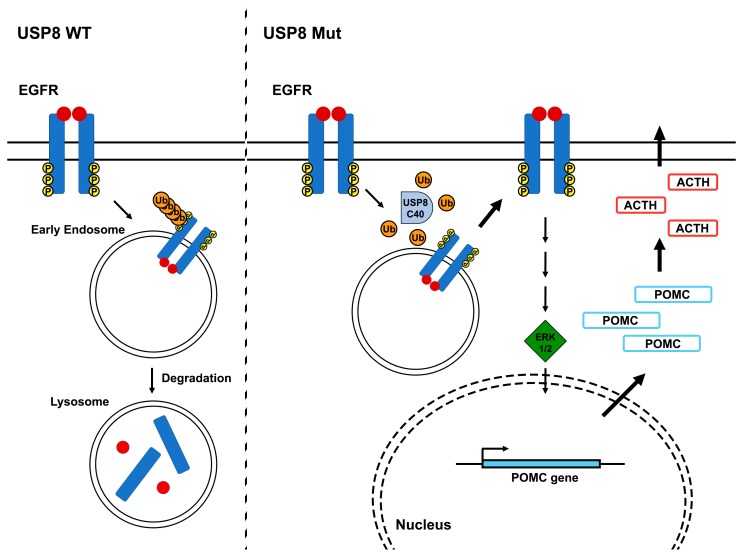
Effects of USP8 gain-of-function on EGFR signaling in ACTH-secreting PitNET. (left panel) After the EGF binding, EGFR undergoes a huge autophosphorylation. This event switches on EGF-induced signaling cascades, but also promotes the recruitment of several ubiquitinases, labeling EGFR for endocytosis, and the lysosomal degradation. (right panel) The mutated USP8 catalytic domain (C40), with its uncontrolled Deubiquitinase catalytic domain (DUB) activity, abrogates the EGFR ubiquitination, inducing its plasma membrane recycling, and permanently boosts EGF signaling cascades switching on proopiomelanocortin (POMC) transcription and ACTH synthesis.

Interestingly, the results of whole exome analyses of corticotrophinomas removed from the pituitary of the CD patients [25,26] indicated that all the mutations of the *USP8* gene sequence were located in or adjacent to the 14-3-3 BM. The most frequent mutations were p.Ser718Pro, p.Ser718del, and p.Pro720Arg:These mutants completely lose the capability to bind to 14-3-3 proteins, showing a higher DUB activity than the WT counterpart [26]. Further analyses revealed that all these USP8 mutants underwent proteolytic cleavage, giving rise to two USP8-fragments (about 90 and 40 kDa, respectively). The 90 kDa fragment covers the region from N terminus to aa 714, whereas 40 kDa fragment includes the sequence from aa 715 to the C terminus (Figure 1). This last fragment contains the complete sequence of the deubiquitinase catalytic domain, but it lacks control of the 14-3-3 proteins, thus acquiring an elevated DUB activity. Since EGFR was previously found to be a target of USP8 deubiquitinase activity and it was deeply involved in ACTH-producing PitNETs, representing a strong promoter of ACTH synthesis [27,28,29,30,31], Reincke et al. examined the effects of USP8 mutations on EGFR regulation and ACTH synthesis. They reported that transfection of USP8 mutants in cultured cells decreases the levels of EGF-induced EGFR ubiquitination [25]. Moreover, USP8 mutants induced both the EGFR retention on the plasma membrane and the recycling of endocytosed EGFR back to the cell surface, causing the continuous activation of EGF signaling (Figure 2) [25]. 

Then, the *USP8* mutation effects were evaluated on ACTH synthesis and secretion. Indeed, the overexpression of USP8 mutants, but not WT form of USP8, increased the *Pomc* promoter activity in an EGFR-transfected cell line. The *Pomc* gene codes for a polypeptide precursor, called proopiomelanocortin (POMC), which is extensively cleaved to ACTH and lipotropin beta (LPH), the major end products. Moreover, treatment of an EGFR-transfected cell line with EGF yielded an increased ACTH production and secretion, and the upregulation of USP8 mutants strengthened the EGF-induced effect (Figure 2) [25]. 

All together these results clearly demonstrate that the USP8 mutations found in the 14-3-3 BM repress the degradation of EGFR, enhancing its accumulation, and consequently, leading to constitutional activation of the EGFR signaling in corticotroph cells. These data are consistent with previous studies that show the key role of EGFR in corticotroph adenomas [27,28,29,30,31] and in the regulation of *POMC* transcription and ACTH production [27,28,29,30,31].

In this context, to better investigate the critical role of EGFR enhanced activity in ACTH-secreting PitNETs, transgenic mice (corti-EGFR-Tg) were generated using the *EGFR* gene under the control of an artificial promoter including pituitary-specific binding sequences of rat *Pomc* transcriptional factors and the *Pomc* enhancer located 7 Kb upstream the transcription start site in order to boost corticotroph-specific human EGFR expression [32].

In this study, 26 corti-EGFR-Tg mice were analyzed by micro magnetic resonance imaging (micro-MRI) at 6.5 and 8 months of age. Pituitary tumors and/or hyperplasia were found in about 65% of the analyzed mice. Moreover, histological analysis revealed characteristic disrupted reticulin fiber network in corti-EGFR-Tg mice pituitary. Interestingly, several tumoral cells displayed an intense CAM5.2 immunoreactivity of the keratin, indicative of Crooke’s hyaline changes, which are features of a severe human CD. These findings were also in agreement with the metabolic dysfunction of the transgenic mice. Indeed, the corti-EGFR-Tg murine model showed an abdominal fat accumulation and augmented body size, an increase in body mass index, and higher plasma glucose levels, all features suggestive of a Cushing metabolic phenotype [32].

Finally, to validate that Cushing phenotype was due to the EGFR upregulation, these transgenic mice were treated with an EGFR inhibitor, such as gefitinib: Treated mice displayed a diminished pituitary tumor size and lower plasma glucose levels. Intriguingly, this study also pointed out the pivotal role of E2F1 in the corticotroph tumorigenesis. In fact, the E2F1 levels were found upregulated in the corticotroph tumor cells, whereas they were abrogated by EGFR inhibition. Moreover, the growth of an ACTH-secreting tumor cell line was deeply reduced by the E2F1 inhibitor HLM 006474. These data are also consistent with other studies showing that E2F1 is required to regulate human POMC and that E2F1 binds to the POMC, binding enhanced by E2F1 phosphorylation [33].

Therefore, the results obtained by the analysis of the corti-EGFR-Tg mouse model support previous findings indicating that EGFR1 activity is mediated by E2F1 and EGFR1 overexpression is associated with aggressive pituitary tumors [32]. Moreover, this study underlines E2F1 increased activity and, then, cell cycle disturbances as critical features of pituitary tumors development also in ACTH tumors, as already largely evidenced in other histotypes. However, the mechanism by which EGFR signaling leads to increase in E2F1 activity is still to be clarified. We can envisage possible mechanisms such as increase E2F1 phosphorylation due to an increased MAP kinase activity, and maybe the induction of the HMGA proteins that has been previously demonstrated to increase E2F1 acetylation levels and frequently associated with cell transformation [34]. 

### 3.1. Clinicopathological Features of ACTH-PitNET Patients Carrying USP8 Mutations

Several studies have been conducted in order to verify whether there were clinicopathological differences between the USP8 mutant and wild-type ACTH-secreting PitNET patients. In a first study, 48 patients, including 18 patients carrying the USP8 mutant form, were retrospectively analyzed after transsphenoidal surgery. It came up that recurrences were more frequent and appeared earlier in patients with USP8 mutant corticotroph tumors [35]. In a further study, no significant differences in hormonal levels and tumoral features in relation to USP8 status were observed analyzing 92 corticotroph patients with 22 cases carrying USP8 mutations. On the contrary, recurrence of CD arose in 23% of USP8-mutated and in 13% wild-type carrying cases, whereas the recurrence-free survival did not significantly change between these two groups [36].

Presence of USP8 mutations has been also investigated in the so-called Nelson’s syndrome (NS), a potentially lethal disease that occurs in bilateral adrenalectomized patients developing ACTH-secreting macroadenomas. Fifteen out of 33 tumors (45%) have been found with *USP8* gene mutations in the exon 14, indicating that the mutated form of USP8 does not drive the corticotroph tumor progression in NS. However, a less favorable outcome has been related to the presence of *USP8* somatic mutations after surgery for NS [37].

### 3.2. USP8 Involvement on other Human Neoplasias: High Expression of USP8 in Lung and Cervical Carcinomas

Recently, it has been reported that both the RNA and protein levels of USP8 were upregulated in cervical squamous cell carcinoma (CSCC) and lung carcinomas compared to normal counterpart tissues [38,39] and high USP8 expression correlated with advanced tumor stage and high recurrence risk in both these carcinomas [40]. Moreover, USP8 was identified as a novel independent prognostic factor for CSCC patients [38]. Interestingly, functional studies showed that USP8 can enhance tumor initiation and progression of lung carcinoma, likely stabilizing receptor tyrosine kinases (RTK), such as EGFR or p-MET, through the interaction with stratifin (SF), a recently identified oncogene [41]. Moreover, the inhibition or the reduced expression of USP8 selectively eliminates gefitinib-resistant and -sensitive non-small cell lung cancer, representing a novel direction for drug development for CSCC and lung cancer therapy [39].

### 3.3. *USP48*, BRAF, and *RASD1* Mutations on ACTH-PitNETs

Interestingly, *USP48*, another deubiquitinase gene, has been found somatically mutated in 21 out 91 ACTH-secreting PitNET patients, carrying a wild-type USP8 [42]. The most prevalent mutations were p.M415I and p.M415V, with a preference for C-T mutations. Moreover, in the same patients, BRAF mutations (encoding p.V600E) were found in 15 tumors. These findings are in agreement with data proving that BRAF improves the transcriptional activity of POMC gene, thus representing a potential mechanism for ACTH overproduction in corticotroph PitNETs, and that BRAFV600E can enhance this effect. The tumor size of the ACTH-secreting PitNETs carrying USP48 or BRAF mutations did not show significant differences in comparison with that of patients carrying wild-type BRAF/USP48. However, patients with BRAFV600E had significantly higher levels of midnight plasma ACTH, confirming also in vivo that BRAFV600E promotes ACTH production. Noteworthily, BRAF and USP48 mutations were not detected in other types of PitNETs, as well as USP8 mutations [43], suggesting that these mutations appear to be unique signatures in corticotroph adenomas. 

Therefore, these results also suggest new therapeutic strategies for ACTH-secreting PitNETs as also supported by the demonstration that primary corticotroph tumor cells carrying BRAFV600E mutation are sensitive to vemurafenib, a BRAF inhibitor. 

Moreover, the presence of frequent USP8, USP48, and BRAF mutations in recurrences of corticotroph PitNETs suggests that hyperproliferation of the corticotroph cells promotes these mutations for some still unknown mechanisms. 

However, the existence of additional mutations involved in ACTH-secreting PitNETs development has also been explored. Recently, a somatic mutation in the gene *RASD1*, which is a component of the corticotropin-releasing hormone receptor signaling system, was identified by utilizing whole-exome discovery sequencing. These results may shed new light on the molecular mechanisms that regulate the corticotropin-releasing hormone receptor, leading to the alteration ACTH production in corticotroph tumors [44,45].

## 4. HMGA Proteins as Drivers of Pituitary Tumorigenesis

The HMGA1a, HMGA1b, and HMGA2 proteins belong to the high mobility group A (HMGA) family. The HMGA1 proteins derive, throughout alternative splicing, from the same *HMGA1* gene positioned on chromosome 6p21, whereas the *HMGA2* gene, that encodes a homologous protein, is located on chromosome 12q13-15. Binding to the minor groove of DNA through their “AT-hook” domains, the HMGA proteins cooperate with the transcription machinery altering the chromatin structure, thus controlling, negatively or positively, the transcriptional activity of numerous genes. HMGA expression levels are particularly low in adult tissues and normal cells, whereas in embryonic cells, as well as in malignant cells, their expression is exceptionally high [46,47]. Indeed, HMGA proteins have a key function during the embryogenesis. In particular, cardiac hypertrophy and type 2 diabetes have been found in *Hmga1*-null mice, underlining the HMGA1 key role in cardiomyocytic development and regulation of the insulin pathway [48]. Conversely, both *Hmga2-*heterozygous and *Hmga2*-null mice showed a pygmy phenotype with a body size diminution of 25% and 60%, respectively, and a strong decrease of fat tissue [49,50]. These data evidence the HMGA2 pivotal function in body size regulation and growth and differentiation of adipocytes. Intriguingly, the critical role of both these genes in the control of the body size is further supported by the phenotype of the *Hmga1*/*Hmga2* double knockout mice that exhibited a very small size (75% smaller than the wild-type mice) likely due to the drastic reduction in E2F1 activity [51].

HMGA overexpression in human cancer is strongly linked to a highly malignant phenotype, since it correlates with a reduced survival and the presence of metastases, thus representing a poor prognostic index [52]. Consistently, several studies have evidenced that HMGA proteins play a critical role in neoplastic transformation. In fact, the silencing of HMGA expression in rat thyroid cells prevented their neoplastic transformation [53]. Moreover, apoptotic cell was induced in anaplastic human thyroid carcinoma-derived cell lines, but not in normal thyroid cells, through the infection with adenoviruses carrying the antisense sequence of the *HMGA1* gene [53]. Moreover, in vivo studies have clearly demonstrated that HMGA overexpression transforms mouse and rat fibroblasts [34] and transgenic mice overexpressing either *HMGA1* or *HMGA2* develop benign and malignant neoplasias [54,55,56,57]. Furthermore, *HMGA2* gene has been found recurrently rearranged in human mesenchymal benign tumors, such as lipomas, lung hamartomas, uterine leiomyomas, endometrial polyps, and breast fibroadenomas following chromosomal translocation regarding the chromosomal region 12q13-15. The chromosomal breaks frequently occur in the third intron of *HMGA2* gene, giving rise to chimeric transcripts constituted of the first three exons of *HMGA2* (encoding the AT-hook domains) and ectopic sequences from other genes. 

Therefore, looking for the oncogenic potential of *HMGA2* overexpression, transgenic mice (Tg-Hmga2) carrying the wild-type *HMGA2* gene under the transcriptional control of the cytomegalovirus promoter that is able to drive a high and essentially ubiquitous expression of the transgene were generated [54]. Intriguingly, Tg-Hmga2 mice showed a giant phenotype together with a predominantly abdominal/pelvic lipomatosis, and they developed Natural Killer T cell lymphomas and prolactin (PRL) and GH secreting- pituitary adenomas [54,55,56,57]. These tumors were detected in the 85% of 6-month-old female Tg-Hmga2 mice with the presence of a large hemorrhagic mass, whereas the transgenic males displayed a lower penetrance (40%) and a longer latency period (≈18 months) [56]. The tumors were composed of three main cell populations: One secreting GH, one secreting PRL, and one secreting both hormones, thus leading to the diagnosis of mixed growth hormone/prolactin cell pituitary adenomas.

Interestingly, *Pit-1*, a gene coding for a pituitary-specific transcriptional factor essential for activation of the *GH* and *PRL* genes, has been found overexpressed in pituitary adenomas, whereas its expression levels were not found in normal adult pituitary glands. Consistently, it has been shown that the HMGA proteins can positively induce PIT1 expression and a positive correlation between *HMGA* and *PIT1* gene expression was found in GH and PRL-secreting PitNETs [58]. Noteworthy, transgenic mice overexpressing *HMGA1* (Tg-Hmga1) showed a similar phenotype.

Therefore, the *HMGA2* transgenic mouse model of pituitary tumorigenesis exhibits several analogies with human PitNETs where there is a female preponderance in pituitary tumor incidence with an earlier onset in female compared to male patients. This similarity between the mouse model and human PitNETs promoted the study of the possible *HMGA2* role in human pituitary tumorigenesis. Intriguingly, it was found that the most recurrent cytogenetic alteration in human PRL-secreting PitNETs was the trisomy of chromosome 12, where the *HMGA2* locus is placed [59]. Moreover, it was reported that the *HMGA2* gene was found amplified in seven of the eight prolactinoma samples examined by dual-color interphase fluorescence in situ hybridization analysis. Simple trisomy, tetrasomy, and chromosomes bearing 12q14–15-derived regions were found as main cytogenetic alterations in the analyzed prolactinomas [59]. This association was, however, detected only in few cases of non-functioning pituitary adenomas (NFPA). 

**Figure 3:**
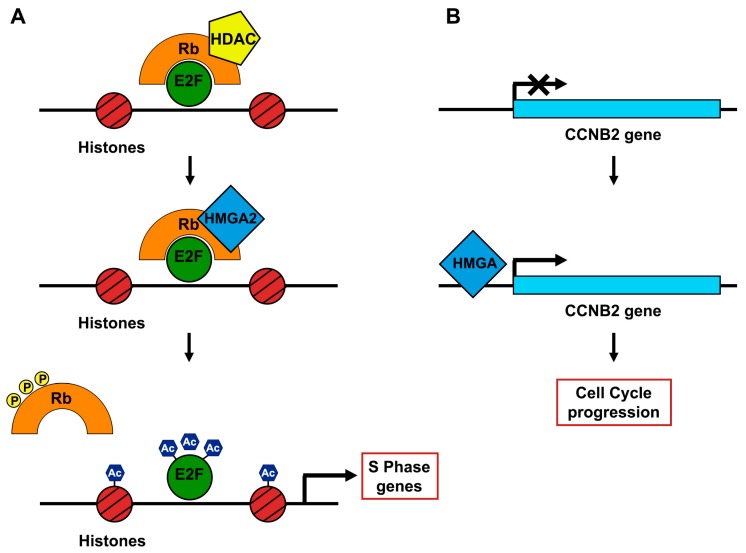
Molecular mechanisms by which the overexpression of HMGA proteins promotes pituitary tumorigenesis. (**A**) HMGA2 displaces HDAC1 from the pRb/E2F1 complex. This induces both histone and E2F1 acetylation, enhancing E2F1 transcriptional activity. (**B**) HMGA proteins bind to CCNB2 promoter inducing its expression and, consequently, the deregulation of cell-cycle.

Therefore, further investigations were aimed to unveil the molecular mechanisms by which HMGA proteins drive pituitary tumorigenesis. A critical mechanism is represented by the ability of the HMGA proteins to promote E2F activity that can be summarized in three steps [60]:

(1) In steady state, pRB and Histone deacetylase 1 (HDAC1) are complexed with E2F1, strongly inhibiting its activity.
(2) HMGA2 displaces HDAC1 interacting with pRB.
(3) The shift of HDAC1 enrolls several enzymes that promote the acetylation of both E2F1 and histones. Thus, E2F1 is activated in its “free” form (Figure 3).

The *HMGA2* enhancer role of E2F1 function in the onset of PitNETs has been further confirmed by crossing Tg-Hmga2 mice with *E2F1* knockout mice [60]. Indeed, the susceptibility to develop PitNETs in the HMGA2^tg^/E2f1^-/-^ mice was almost totally rescued, indicating that the E2F1 activation triggered by HMGA2 is a crucial step in pituitary tumorigenesis. However, the HMGA2^tg^/E2f1^-/-^ mice showed a notable pituitary hyperplasia, suggesting that the *HMGA2* gene is involved in several molecular mechanisms, other than E2F1 activation, inducing pituitary tumorigenesis. These results are perfectly consistent with the numerous roles acted by the *HMGA* genes in human tumorigenesis. Noteworthily, the expression profile of PitNETs induced in HMGA transgenic mice showed the overexpression of the *ccnb2* gene, which encodes the cyclin B2 protein, a master controller of the cell cycle. Moreover, functional studies also confirmed that HMGA proteins can induce a ccnb2 positive regulation. Accordingly, a positive correlation between CCNB2 expression and both HMGA1 and HMGA2 levels in different histotypes of human PitNETs has been described [34]. 

The HMGA2 ability to accelerate the cell cycle by enhancing E2F activity and promoting cyclin B expression is in agreement with former data that have reported the development of pituitary tumors in engineered mouse models bearing mutation in cell cycle master regulator genes, such as p27^Kip1^ and RB [61]. Accordingly, cyclin/cyclin-dependent kinase (Cdk) complexes, including cyclin D-binding Cdk4 and its inhibitor INK4 as well as cyclin E-binding Cdk2 and its inhibitor p27^Kip1^, have been found altered in human tumors displaying cell-cycle deregulation [61].

### 4.1. HMGA Overexpression Correlates with a More Aggressive Phenotype of PitNETs

Two different studies describe a correlation between HMGA overexpression and a more aggressive phenotype. In particular, HMGA2 upregulation was frequently reported in several PitNETs subtypes; such as prolactin, adrenocorticotrophic hormone, or follicle-stimulating hormone/luteinizing hormone, and in null cell PitNETs. The expression of both HMGA1 and HMGA2 was significantly increased in aggressive adenomas or macroadenomas compared to in non-aggressive ones. Furthermore, the expression levels of HMGA proteins displayed the highest upregulation in the most aggressive PitNETs (grade IV), compared to grades I, II, and III, showing, consistently, a direct correlation with the levels Ki-67, a widely used proliferation marker [62,63].

Recently, novel experimental models suggest a pivotal role of the HMGA protein overexpression in the progression step of pituitary tumorigenesis. Indeed, mice overexpressing *Hmga2* (Hmga2/T) were crossed either with the knock-in mice for the Cdk4^R24C^ mutation, an engineered mouse strain that bears a single point mutation (R24C) in the first exon of the mouse *Cdk4* gene, making Cdk4 protein unresponsive to INK4 inhibitors, or with the knockout mice for p27^kip1^ (p27-KO) [64].

Both the obtained double mutant mice (Hmga2/T;p27-KO and Hmga2/T;Cdk4^R24C^) displayed a significantly earlier onset of more aggressive pituitary tumors, with increased features of malignancies such as the upregulation of Ki-67 and a boosted number of mitoses. Moreover, in several cases, the presence of malignant cells justified the diagnosis of pituitary carcinoma [64].

### 4.2. Regulation of HMGA Expression by Non-Coding RNAs in Pituitary Tumors

As discussed before, the overexpression of the HMGA proteins does not appear always associated with the amplification of their genomic loci. Therefore, other mechanisms, such as epigenetic events involving the non-coding RNAs, have been taken in consideration. Indeed, over the last 15 years, large-scale genomic technologies (high-resolution microarray, whole genome, and RNA sequencing) combined with bioinformatic approaches have allowed an extensive analysis of the human transcriptome, revealing that only a small amount (about 2%) of the genomic DNA actually encodes proteins, while a large number of transcripts are not translated into proteins [65,66]. These RNA molecules, named non-coding RNAs (ncRNAs), seem to play a crucial role in a variety of cellular and physiologic functions. 

NcRNAs are generally classified into two categories according to their length. The size of small ncRNAs, such as miRNAs, is typically less than 200 nucleotides (nt) and they include miRNAs, small nucleolar RNAs (snoRNAs), and piwi RNAs (piRNAs), while the long non-coding RNAs (lncRNAs) are typically greater than 200 nt and comprise multiple members with different functions and complexity. 

MiRNAs are small non-coding RNAs consisting of about 22 nucleotides in their mature form. Mature miRNAs are capable of inducing gene silencing by repression of protein synthesis or by degradation of messenger RNAs. Each miRNA is able to bind multiple mRNAs and, in turn, each mRNA can be regulated by multiple miRNAs cooperatively [67]. The repression of miRNA-mediated target genes occurs through perfect or imperfect recognition of miRNA recognition elements (MRE) in the 3 ‘UTR (untranslated regions) of the target mRNAs [68]. 

### 4.3. MiRNAs Targeting the *HMGA* Genes

**Figure 4:**
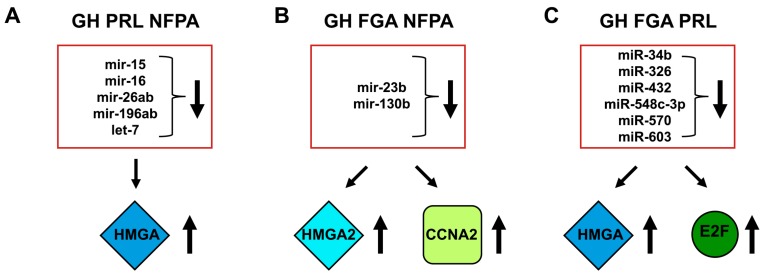
HMGA targeting miRNAs in PitNETs. (**A**, **B**, and **C**). Deregulated miRNAs in pituitary tumorigenesis. Several miRNAs are able to target other cancer-related genes (CCNA2 and E2F1).

By using bioinformatic tools for the prediction of miRNA-targeting sites, five miRNAs (miR-15, miR-16, miR-26ab, miR-196ab, and Let-7) showing MRE sites on the 3′UTRs of *HMGA1* and *HMGA2* have been identified. Then, the ability of these miRNA to downregulate both HMGA1 and HMGA2 protein expression was validated. Interestingly, the expression levels of these miRNAs were strongly downregulated in all the analyzed PitNETs of different histotypes, and their expression was inversely correlated with that of the *HMGA* genes [69] (Figure 4). Subsequently, it was shown that miR-23b and miR-130b, able to target *HMGA2* and *CCNA2*, respectively, were drastically downregulated in GH, gonadotroph (FGA) and NFPA adenomas in comparison with normal pituitary gland and correlated with their respective targets. Interestingly, the overexpression of such miRNAs inhibited cell proliferation, arresting the cells in the G1 and G2 phase of the cell cycle [70] (Figure 4).

**Figure 5:**
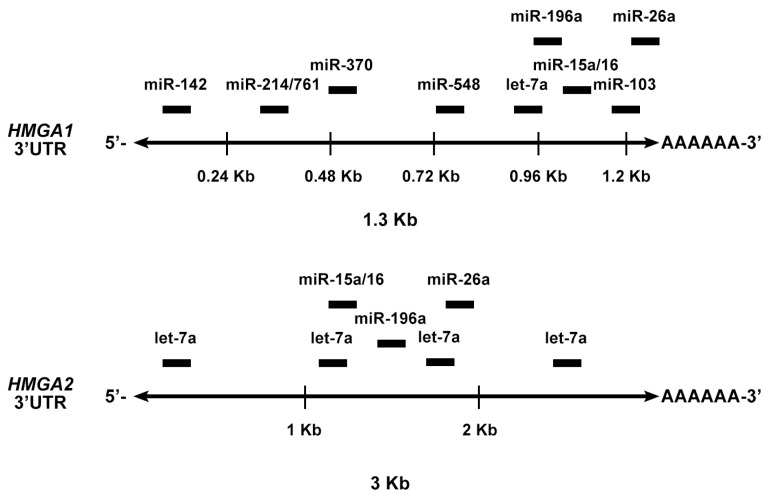
Binding sites of HMGA1/2-targeting miRNAs. Schematic representation of human HMGA1 and HMGA2 3′UTRs and the relative positions of some predicted binding sites for HMGA-targeting miRNAs.

Furthermore, the miRNA expression profile of 12 somatotroph adenomas by microarray analysis, identified 19 miRNAs as differentially expressed between pituitary adenomas and normal pituitary. The focus of the study was on eight of the down-regulated miRNAs (miR-34b, miR-326, miR-432, miR-548c-3p, miR-570, and miR-603) (Figure 5), because they were predicted, and then validated, to target *HMGA1*, *HMGA2*, and/or *E2F1* genes. Interestingly, these miRNAs were also found downregulated in PRL and FGAs, suggesting that their downregulation was a general event in pituitary tumorigenesis. Enforced expression of the downregulated miRNAs had a negative role on the growth regulation of pituitary adenoma cells. Accordingly, an inverse correlation was found between their expression and HMGA1 and HMGA2 protein levels in GH adenomas, suggesting that the reduced levels of these miRNAs could contribute to the HMGA-mediated pituitary tumorigenesis [71] (Figure 4).

### 4.4. Deregulated Expression of lncRNAs Controlling HMGA Expression Levels in PitNETs 

Interestingly, HMGA overexpression in PitNETs also takes the contribution of a deregulated expression of the lncRNAs, a class of transcribed RNA molecules that also includes the pseudogenes and ranges in length from 200 nt to ~100 kilobases (kb) with no protein-coding ability. Pseudogenes are a class of RNA molecules that have lost their coding potential because of premature or delayed stop codons, deletions/insertions, and frameshift mutations that abrogate translation into functional proteins [72,73].

It has been reported that two recently identified HMGA1-pseudogenes, *HMGA1P6* and *HMGA1P7*, may play a role in PitNETs development protecting, by a ceRNA-sponge mechanism, *HMGA1, HMGA2,* and other cancer-related gene mRNAs from miRNAs able to target their 3′UTR [74,75,76,77,78]. They are overexpressed in NFPA, GH, and FSH-LH-secreting adenomas and their expression positively correlates with that of HMGA1 and HMGA2. Finally, functional studies have also demonstrated that both pseudogenes increase proliferation and migration of the pituitary adenoma cell line AtT20, indicating that their overexpression contributes to pituitary tumorigenesis [79].

Very recently, another lncRNA, the cancer susceptibility candidate 2 (CASC2), able to decrease *HMGA2* expression, shows a reduced expression in various PitNET histotypes. Moreover, CASC2 inhibits growth and invasion of AtT-20 and GT1-1 cells and xenograft tumor growth likely playing a role in pituitary tumorigenesis [80].

Finally, the analysis of the lncRNA expression profile of 12 FGAs revealed 1467 upregulated and 1909 downregulated lncRNAs. Among them, the RPSAP52 (ribosomal protein SA pseudogene 52) gene was one of the most upregulated. Interestingly, it represents the lncRNA antisense of the *HMGA2* gene, and its expression positively correlates with the expression of HMGA2 and HMGA1. RPSAP52 expression leads to increased HMGA1 and HMGA2 protein levels, and, consistently, enhances cell growth and cell cycle progression at the G1-S transition step by acting as a sponge for miRNAs (miR-15a, miR-15b, and miR-16). Therefore, RPSAP52 may be considered a novel player in the development of human PitNETs by enhancing the expression of the HMGA1 and HMGA2 proteins [81].

## 5. Deregulated Expression of other miRNAs in Pituitary Tumorigenesis

In the last years, several genome-wide miRNA expression profiles were performed in pituitary adenomas and versus normal pituitaries in order to identify a pituitary adenoma-specific miRNA signature. 

Mao et al. compared 52 GH-secreting pituitary tumors versus normal glands, and they found 23 upregulated miRNAs and 29 downregulated miRNAs. Interestingly, miR-126 and miR-381, which were found downregulated, target the *PTTG1* (pituitary tumor-transforming 1) gene, which codes for securin, an oncogene with an important role in the proliferation of pituitary cells [82]. *PTTG1* was demonstrated to be a target also of miR-655, miR-300, miR-381, and miR-329, whose overexpression was able to suppress the viability and proliferation of human pituitary tumor cells [83].

Butz et al. examined the expression of Wee1, a cell cycle regulator that inhibits Cdk1, thereby preventing the entry of the cells into mitosis at the G2/M checkpoint. The authors identified that three miRNAs (miR-128a, miR-155, and miR-516a-3p) are able to inhibit Wee1 protein expression and then cell proliferation [84].

Trivellin et al. have demonstrated that miR-107 targets AIP gene coding for cytoplasmic cochaperone protein regulating cell proliferation via the cAMP pathways and is involved in the predisposition to develop FIPA syndrome and in sporadic somatotroph adenomas. It has been suggested that miR-107, overexpressed in pituitary adenomas, may act as a tumor suppressor-miR targeting the AIP gene [85]. 

By evaluating the miRNA expression profile of bromocriptine-resistant versus bromocriptine-sensitive prolactinomas, it was found that miR-93 and miR-126 were upregulated in bromocriptine-resistant prolactinomas, and their silencing significantly increased the sensitivity of cells to dopamine agonist treatment. Moreover, miR-93 was demonstrated to induce cell proliferation and tumor development by targeting p21^WAF^^1/CIP1^, a cyclin kinase inhibitor [86].

Recently, a miRNA expression profile of 12 FGAs revealed 57 downregulated and 44 upregulated miRNAs in comparison with the normal gland. MiR-410 was one of the most downregulated miRNAs, and it was previously reported to act as tumor suppressor. *CCNB1* gene, coding for cyclin B, an important cell cycle regulator, has been demonstrated to be a target of miRNA-410 and that the restoration of miRNA-410 expression in pituitary adenoma cells resulted in the inhibition of cell proliferation. In the same study, miR-410 was predicted to target also cyclin A, cyclin D and CDKs that have a critical role in cell cycle regulation. Therefore, miR-410 downregulation might contribute to the progression of pituitary adenomas increasing cyclin levels and, thereby, enhancing the G1-S phase of the cell cycle [79].

In order to identify miRNAs able to distinguish the invasive pituitary adenomas from the non-invasive ones, Yu et al. demonstrated that the expression of miR-24, miR-93, miR-126, and miR-34a was significantly decreased in invasive compared with non-invasive pituitary tumors [87].

## 6. Circular RNAs Associated with miRNAs in NFPA 

Increasing evidences indicate that circular RNAs (circRNAs) can influence miRNA transcriptional activity by harboring abundant conserved MREs [87]. It has been reported that miR-145-5p targets the translationally controlled tumor protein (TPT1), and was markedly decreased in NFPAs samples, and negatively correlated with tumor invasiveness. Moreover, circOMA1, a novel circular RNA, functions as sponge for miR-145-5p, then promoting NFPA progression [88]. 

## 7. Other lncRNAs Involved in Pituitary Tumorigenesis

The maternally expressed gene 3 (MEG3) represents the first recognized lncRNA tumor suppressor in pituitary adenomas [89]. MEG3 is an imprinted gene, belonging to the DLK1-MEG3 locus positioned on chromosome 13q32 [90], and is expressed only from the allele on the maternal chromosome. It was found completely silenced in NFPAs by promoter methylation [91]. Experimental studies have demonstrated that MEG3 restoration induced G1 arrest and suppressed xenograft tumor growth in vivo in nude mice. 

Conversely, increased expression of the lncRNA actin filament-associated protein 1 antisense RNA 1 (AFAP1-AS1) was found in pituitary tumors. This is a recently recognized cancer-related lncRNA deriving from the antisense strand of DNA at the AFAP1 coding gene locus. Higher levels of AFAP1-AS1 in pituitary tumor tissues compared to the adjacent ones have been observed. Additionally, knockdown of AFAP1-AS1 inhibited proliferation of pituitary cell lines, determined cell cycle arrest in the G1-S transition phase, and enhanced apoptosis in these cells by regulation of the PTEN/PI3K/AKT signaling pathway [92].

Interestingly, the deregulation of some lncRNAs seems associated with an invasive phenotype of PitNETs. Indeed, significantly increased expression of Hox transcript antisense intergenic RNA (HOTAIR), a lncRNA involved in cancer progression by remodeling the chromatin landscape, was found in invasive and non-invasive NFPAs compared to normal anterior pituitary and its expression correlated with invasive biological behavior and larger tumor size [93]. HOTAIR interacts with polycomb repressive complex 2 (PRC2), which enhances H3K27 trimethylation to decrease expression of multiple genes [94,95]. 

Another lncRNA correlated with an invasive behavior of PitNETs is represented by C5orf66-AS1 that was found significantly downregulated in pituitary null cell adenoma tissues compared with normal pituitary tissues and more frequently in invasive adenomas than in non-invasive adenomas. C5orf66-AS1 inhibited cell viability and invasion overexpression of GT1-1 cells, supporting its negative role in the development and invasion of pituitary null cell adenomas [96].

Furthermore, the oncofetal lncRNA H19 a maternally expressed and paternally imprinted gene, reciprocally imprinted and regulated with its neighboring gene IGF2 [97], is expressed at significantly higher levels in invasive and aggressive GH-secreting PitNETs compared to non-invasive ones [98]. Intriguingly, it has been reported that the *HMGA1P7*, which has been found upregulated in several PitNET histotypes, including the GH-secreting ones, may act as decoy for the H19-targeting miRNAs [77], thus suggesting that *HMGA1P7* may have a role in pituitary tumorigenesis also by enhancing H19 expression levels through a ceRNA mechanism. 

## 8. Conclusions and Perspectives

The involvement of the USP8 mutations in the pathogenesis of ACTH-secreting PitNETs constitutes one of the most stimulating discoveries in both PitNETs and CD fields. However, several questions are still open. Indeed, the molecular mechanisms responsible for the specificity of these mutations for ACTH-secreting PitNETs is already completely unknown since no mutations have been detected in more than 200 PitNETs subtypes including GH, PRL, and NPFA tumors. Moreover, the validation of the oncogenic role of the USP8 mutations has not been reported yet: The generation of transgenic mice carrying a mutated USP8 gene under the transcriptional control of a corticotroph specific promoter would answer this question. Conversely, the corti-EGFR-Tg mice [32] validate the oncogenic potential of EGFR overexpression in pituitary tumorigenesis representing, moreover, an excellent model for new therapeutic strategies. 

Another important point that comes from the recent studies on the molecular mechanisms underlying the development of PitNETs is the oncogenic function of HMGA in pituitary tumorigenesis. Indeed, this role has been clearly validated by the development of GH/PRL-secreting tumors in transgenic mice overexpressing either *HMGA1* or *HMGA2*, also suggesting their function as potential first driver in this neoplastic process. 

The critical role of HMGA proteins in PitNETs development and the regulation of their expression by epigenetic mechanisms opens the perspective of different innovative approaches to the therapy of PitNETs resistant to the conventional treatment. Indeed, it would be possible to treat the PitNETs with drugs able to impair the HMGA function, such as trabectedin, recently reported to act by inhibiting the binding of the HMGA proteins to their responsive promoters [99]. Moreover, this treatment could be combined with the restoration in the PitNET tissues of the miRNAs able to target the *HMGA* mRNAs.

Finally, the Tg-HMGA mice that develop PitNETs along with different aggressivity when crossed with mice carrying the deletion of the p27^kip1^ gene or a Cdk4^R24C^ mutation, represent an excellent model for the validation of innovative or conventional therapies. In fact, it has been already reported that SOM230 induced a drastic regression of the tumor in Tg-Hmga2 mice [100].

Therefore, the recent research advances that highlight the role of USP8 mutations, HMGA expression, and ncRNA deregulation in PitNETs enormously enhance the comprehension of the mechanisms of pituitary tumorigenesis and open new perspectives of a more effective therapy of these neoplasias.
